# Fighting COVID-19 with water

**DOI:** 10.7189/jogh.10.010344

**Published:** 2020-06

**Authors:** Gordan Lauc, Alemka Markotić, Ivan Gornik, Dragan Primorac

**Affiliations:** 1 University of Zagreb Faculty of Pharmacy and Biochemistry & Genos Glycoscience Research Laboratory, Zagreb, Croatia; 2 Clinics for Infective Diseases, Dr. Fran Mihaljević, Zagreb, Croatia; 3 Department of Emergency Medicine, Clinical Hospital Zagreb, Zagreb, Croatia; 4 St Catharine Hospital, Zagreb, Croatia; 5 Eberly College of Science, Penn State University, USA; 6 University of Split School of Medicine, Split, Croatia; 7 University of Osijek School of Medicine, Osijek, Croatia; 8 University of Osijek Faculty of Dental Medicine and Health, Croatia; 9 Medical School REGIOMED, Coburg, Germany

Recent epidemiological data from several sources show that transmission of coronavirus disease (COVID-19) is more efficient in cold and dry climate than in warm and humid locations [1,2]. Strong seasonal character of most respiratory viruses also suggests the important role of environment for viral transmission [3]. This is most often interpreted by seasonal indoor crowding and effects of temperature and humidity on stability of viral particles, but we suggest that this effect is by large a consequence of inactivation of the mucosal barrier by dry air in heated indoor spaces.

Inter-individual differences within the human population are glycan-based in a substantial part and glycan diversity represent one of the main defenses of all higher organisms against pathogens. Glycans (which are covalently attached to most proteins) are chemical structures that are being inherited as complex traits, which enables diversity and results in significant inter-individual variation in glycome composition [4]. Most interactions between humans and pathogens involve glycans, and diversity in the thick glycocalyx layer that covers the cell membrane provides us with the important “herd innate protection” [5]. Additional layer of protection is provided by mucins, a group of highly glycosylated proteins that are secreted onto our mucosal barriers. Mucins mimic cell surface glycosylation and by acting as a decoy trap viral particles, which are then transported out of airways by mucociliary clearance [6].

However, for this barrier to stay functional it is necessary to stay well hydrated to both maintain its structural integrity and enable constant flow of mucins that carry viruses and other pathogens out of the airways [6]. If exposed to dry air, these barriers dry out and cannot perform their protective functions [7]. This promotes both initial infection and expansion of viruses along the airways [8]. Animal experiments convincingly demonstrated that increasing relative humidity from 20% to 50% can significantly decrease mortality from influenza infections [8]. The Yale research team confirmed that low humidity influences immune response. It does so through preventing cilia from removing viral particles and mucus, reducing ability of airway cells to repair mutilation caused by virus in the lung and failing to alert neighbouring cells by virus-infected cells via interferons to the viral threat [8].

The fact that both bacteria and viruses are less infective in moist environment argues against the effect of humidity on stability of virus particles. It suggests that protective effects of humidity on mucosal barrier may be a dominating element. The same conclusion came from Noti and colleagues who concluded that indoor relative humidity of >40% will significantly reduce the infectivity of aerosolized influenza virus particles [9].

Dehydration of mucosal barriers is frequent in heated spaces and may be one of the main reasons why respiratory infection show significant seasonality. Cold air has very low capacity to dissolve water. When cold air from outside enters a room, its relative humidity becomes very low. For example, air with 100% humidity at 5°C when heated to 25°C will have relative humidity of approximately 20%. Several studies in the US indicated that relative humidity in both residential [10] and commercial spaces [11] in US is below 25%, which is very low and results in dehydration of mucosal barriers and enhanced viral transmission. A similar problem may also occur also in warm periods, when excessive cooling with limited exchange of air can also result in very low indoor air humidity. It is generally recommended that relative humidity in living and working environments should be around 45% [3,12]. Since damaged mucosal membranes (ie, during viral infection) have impaired secretion capacities, room humidity for respiratory patients may need to be even higher.

In addition to being a protection against initial infection, functional mucosal barrier is also important in suppression of viral progression in already infected patients. Since many hospitals have very dry air, providing humidified air to patients in early stages of the disease may be beneficial. Dry air may be particularly relevant for patients in need of mechanical ventilation since many adult ventilators (in particular in auxiliary hospital units) do not have active humidification. Prolonged inhalation of very dry air is known to promote lung inflammation and humidification of ventilated air is recommended since it may reduce lung inflammation [13]. However, there are some contraindications in patients with low tidal volumes like those with acute respiratory distress syndrome and patients with high minute ventilations volumes [14]. In the current situation of the COVID-19 pandemic with numerous patients on mechanical ventilation, it would be important to investigate the potential benefit and risks of humidification in patients with COVID-19.

**Figure:**
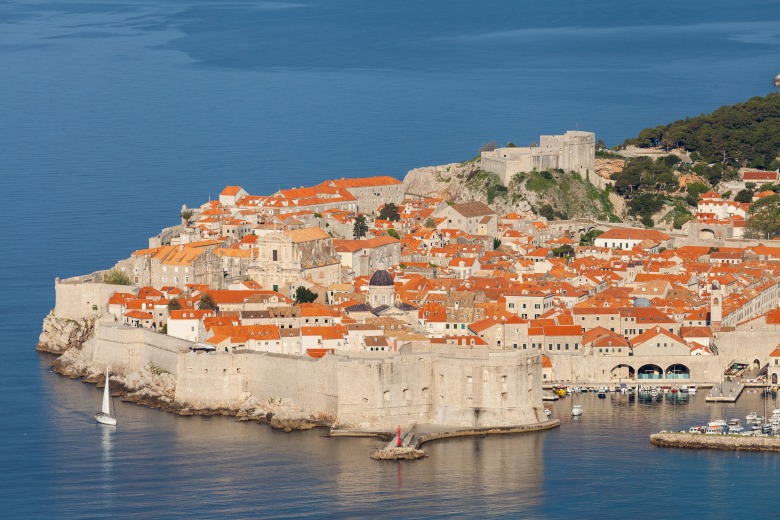
Photo: Mucosal barrier covering our epithelia functions as a protective shield against pathogen invasion in a way that is very similar to walls and a moat that were protecting medieval cities like Dubrovnik. Dehydration of this thick glycan layer inactivates this first line of defence and promotes both initial infection and subsequent expansion of the virus through airways (City of Dubrovnik, Diego Delso, delso.photo, License CC-BY-SA, via Wikimedia Commons).

It was reported that during Covid-19 outbreak in Wuhan many health care workers got infected. Similar situation is repeating in Italy and New York, which is decreasing the capacity of the health care system. Considering the evident detrimental effect of dry air on our mucosal barrier and its role of the first line of defence against infection [15], in situation of rapidly progressing COVID-19 pandemics it would be essential to aggressively promote active re-humidification of dry air in all public and private heated spaces. Furthermore, wherever possible patients on ventilators should be ventilated with humidified air.
